# Dataset of spiking and LFP activity invasively recorded in the human amygdala during aversive dynamic stimuli

**DOI:** 10.1038/s41597-020-00790-x

**Published:** 2021-01-14

**Authors:** Tommaso Fedele, Ece Boran, Valerii Chirkov, Peter Hilfiker, Thomas Grunwald, Lennart Stieglitz, Hennric Jokeit, Johannes Sarnthein

**Affiliations:** 1 National Research University Higher School of Economics, Moscow, Russian Federation; 2 Klinik für Neurochirurgie, UniversitätsSpital Zürich und Universität Zürich, Zurich, Switzerland; 3 Lomonosov Moscow State University, Moscow, Russian Federation; 4 Schweizerische Epilepsie-Klinik, Zurich, Switzerland; 5 Zentrum für Neurowissenschaften Zürich, Zurich, Switzerland

**Keywords:** Perception, Amygdala, Neuronal physiology

## Abstract

We present an electrophysiological dataset collected from the amygdalae of nine participants attending a visual dynamic stimulation of emotional aversive content. The participants were patients affected by epilepsy who underwent preoperative invasive monitoring in the mesial temporal lobe. Participants were presented with dynamic visual sequences of fearful faces (aversive condition), interleaved with sequences of neutral landscapes (neutral condition). The dataset contains the simultaneous recording of intracranial EEG (iEEG) and neuronal spike times and waveforms, and localization information for iEEG electrodes. Participant characteristics and trial information are provided. We technically validated this dataset and provide here the spike sorting quality metrics and the spectra of iEEG signals. This dataset allows the investigation of amygdalar response to dynamic aversive stimuli at multiple spatial scales, from the macroscopic EEG to the neuronal firing in the human brain.

## Abstract (summary)

**Table:** 

Measurement(s)	brain electrical activity • response to aversive stimulus • medial temporal lobe • neuronal action potential • functional brain measurement • response to stimulus • amygdala
Technology Type(s)	Electrocorticography • microelectrode
Factor Type(s)	aversive vs neutral stimulus • seizure onset zone
Sample Characteristic - Organism	Homo sapiens

Machine-accessible metadata file describing the reported data: 10.6084/m9.figshare.13292561

## Background & Summary

Several aspects of perception and cognition involve the amygdala. Neural activity within the amygdala is implied in novelty detection^1^, perception of faces^2^, emotions^3^ and aversive learning^4^. Emotional recognition is facilitated by presentation of fearful facial expression and especially their dynamic presentation^5^. Presentation of dynamic faces has been shown to elicit strong electrophysiological responses in the scalp electroencephalography (EEG)^6^. Within the face perception network, the human amygdala is an important node^7^ where its role has been mainly investigated by means of Blood Oxygen Level Dependent (BOLD) responses^8–10^. Electrophysiological oscillatory responses in the human amygdala are mostly explored in patients with refractory epilepsy who are undergoing pre-surgical monitoring^11,12^. In these patients, the intracranial electroencephalography (iEEG) records local field potentials that result from the activity of thousands of neurons^13^. Thanks to technological advance, iEEG can be combined with recordings of single neuron activity in the human amygdala^14–16^. While these two different types of data provide complementary information on the processing of sensory stimuli, their simultaneous recording remains rare.

**Fig. 1:**
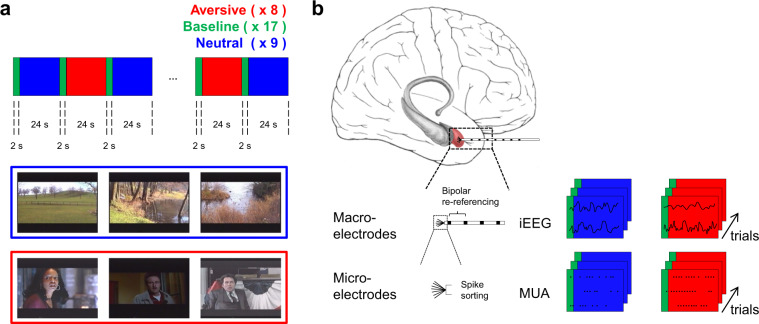
Task and recordings. (**a**) Aversive (red) and neutral (blue) video sequences were presented in blocks of 24 s, interleaved with a repeated 2 s neutral baseline (green). The images are representative video stills drawn from the video sequences. (**b**) iEEG was recorded with macroelectrodes and neuronal action potentials were recorded with microelectrodes. iEEG signals were stored trial-wise as recorded and after bipolar re-referencing. Neuronal action potentials (spikes) were extracted from the microelectrode recordings by spike sorting and stored trial-wise for each neuron.

Here, we describe a publicly released data set recorded from 14 amygdalae of 9 epilepsy patients. It consists of simultaneously acquired iEEG and single-neuron recordings. Differences in amygdala activation were found in response to watching sequences from thriller and horror movies showing actors portraying fearful faces in contrast to relaxing landscape recordings. The task (Fig. 1a) presents salient visual stimuli in a naturalistic way, different from most human single-neuron studies that have only used static stimuli^3,16,17^. Previous publications with the same task have shown strong amygdala responses with BOLD^10,18^ and iEEG^11,12^ together with enhanced firing of single neurons^12^. In a detailed analysis of the dataset^12^, we have described the interactions between iEEG and neuronal firing. Along with the iEEG traces and neuronal recordings, we here provide the technical validation of the quality of the isolated neurons, the localization information for iEEG electrodes, and the task video^19^. This dataset represents a unique opportunity for further investigation of the cross-scale dynamics that define the relation between macroscopic oscillatory activity in the iEEG and the neuronal firing in the human amygdala.

## Methods

### Task

Short video sequences with dynamic fearful faces were compiled to activate the amygdala (Fig. 1a). The video was first used with fMRI in^10^ and later with iEEG^11^ and single neuron recordings^12^. The video is available in the original AVI format and read by a custom program^19^. Schacher *et al*.^10^ describe the video as follows: “To activate the amygdala, we developed a paradigm utilizing visual presentations of dynamic fearful faces. Stimuli were presented in a block design. The paradigm consisted of eight activation (aversive) and eight baseline (neutral) blocks each lasting 24 seconds. The activation condition consisted of 75 brief episodes (2 to 3 seconds) from thriller and horror films. All episodes showed the faces of actors who were expressing fear with high intensity. None of the episodes showed violence or aggression. Quality and applicability of film sequences were evaluated by an expert panel consisting of nine psychologists. Of an initial collection of 120 scenes, only sequences that were considered appropriate by the majority of the expert panel were extracted for the paradigm. Evaluation criteria were as follows: 1) actor’s face is clearly visible; 2) emotion displayed is clearly recognizable as fear; 3) fear is the only clearly recognizable emotion (no other emotion, e.g., anger, sadness, surprise, is displayed); and (4) the fear displayed is of high intensity. During baseline blocks, 72 short episodes of similar length (2 to 3 seconds) with dynamic landscape video recordings were presented. Video clips of calm domestic landscapes were used owing to their stable low emotional content while their general visual stimulus properties were comparable with the movie clips. Frequency and duration of the sequences (2 to 3 seconds) were matched for aversive and neutral conditions.”

### Participants

Nine participants participated in the study (Table 1). All participants were patients with drug-resistant focal epilepsy. They were implanted with depth electrodes in the amygdala and in contiguous areas of the mesial temporal lobe for the potential surgical treatment of epilepsy. The implantation sites were selected solely based on the clinical indication. The study was approved by the institutional ethics review board (Kantonale Ethikkommission Zürich, PB-2016-02055). All participants provided written informed consent to participate in the study. The ethics approval covers the administration of multiple cognitive tasks. Some participants participated in several cognitive tasks. The data obtained in one of these other cognitive tasks has already been analysed and published earlier^20,21^.

**Table 1:** Participant characteristics.

Participant number	Age	Gender	Pathology	Implanted electrodes	Seizure onset zone (SOZ) electrodes
1	31	M	sclerosis	AL, AR	AR
2	48	M	gliosis	AL, AR	AR
3	19	F	sclerosis	AL	
4	22	M	sclerosis	AL	
5	34	M	sclerosis	AR	AR
6	23	M	sclerosis	AL, AR	AR
7	39	M	gliosis	AL, AR	AR
8	27	F	astrocytoma	AL	
9	22	M	sclerosis	AL, AR	AL, AR

M: male; F: female; A: amygdala; L: left; R: right.

### Recording setup

Data were recorded with a standard setup used in many hospitals that do human iEEG and single neuron recordings. We replicate here the description given in our earlier publications^19–21^. “We measured iEEG with depth electrodes (1.3 mm diameter, 8 contacts of 1.6 mm length, spacing between contact centers 5 mm, ADTech®, Racine, WI, www.adtechmedical.com), implanted stereotactically into the amygdala. Each macroelectrode had nine microelectrodes that protruded approximately 4 mm from its tip (Fig. 1b). Recordings were done against a common intracranial reference at a sampling frequency of 4 kHz for the macroelectrodes and 32 kHz for the microelectrodes via the ATLAS recording system (0.5–5000 Hz passband, Neuralynx®, Bozeman MT, USA, www.neuralynx.com). iEEG data were resampled at 2 kHz.” In the presented dataset, we share epoched iEEG data (trials of 26 seconds) as recorded and after bipolar re-referencing, and neuronal activity in the form of time stamps and average neuronal spike waveform.

### Depth electrode localization

Electrodes were localized in the same way as in our earlier publications^19–21^, which we replicate in the following. “We used postimplantation CT scans and postimplantation structural T1-weighted MRI scans. Each scan was aligned to the ACPC (anterior commissure, posterior commissure) coordinate system. For each participant, the CT scan was registered to the postimplantation scan as implemented in FieldTrip^22^. In the coregistered CT-MR images, the electrode contacts were visually marked. The contact positions were normalized to the MNI space and assigned to a brain region using Brainnetome^23^. Anatomical labelling of each electrode contact was verified by the neurosurgeon (L.S.) after merging preoperative MRI with postimplantation CT images of each individual participant in the plane along the electrode (iPlan Stereotaxy 3.0, Brainlab, München, Germany). We specify whether electrodes were inside the seizure onset zone (SOZ).”

### Spike detection and neuron identification

**Fig. 2:**
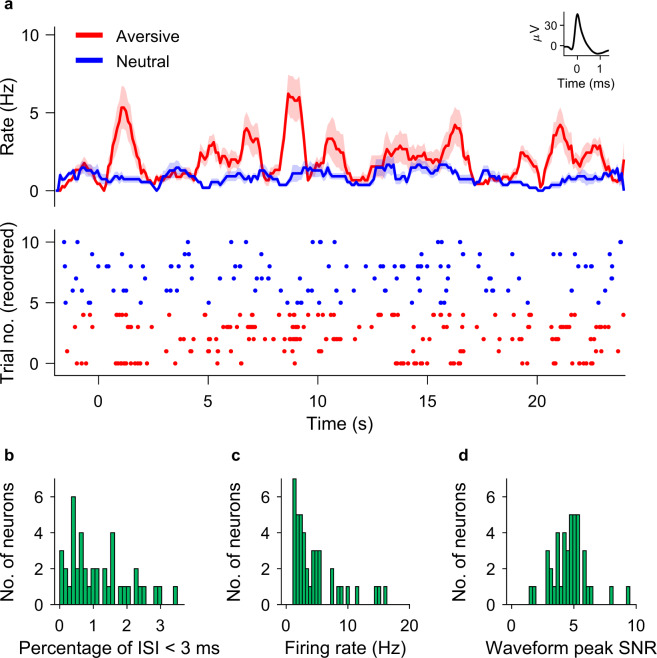
Neuronal firing and spike sorting quality metrics. (**a**) Example neuron in the amygdala. Top: Peristimulus time histogram (bin size: 100 ms; step size: 10 ms) for aversive (red) and neutral (blue) conditions. Shaded areas represent ± s.e.m. across trials of all spikes associated with the neuron Inset: mean extracellular waveform ± s.e.m. Bottom: Raster plot of trials reordered by trial condition for plotting purposes only. The trial onset is at time t = 0. (**b**) Histogram of percentage of inter-spike intervals (ISI) <3 ms. The majority of neurons had less than 0.5% of short ISI. (**c**) Histogram of average firing rate for all neurons. (**d**) Histogram of the signal-to-noise ratio (SNR) of the peak of the mean waveform.

For spike sorting, we followed the same procedure as in our earlier publications^19–21^, where we described the method as follows. “The Combinato package (https://github.com/jniediek/combinato) was used for spike sorting^24^. Combinato follows a similar procedure to other freely available software packages: peak detection in the high-pass (>300 Hz) signal, computation of wavelet coefficients for detected peaks, and superparamagnetic clustering in the feature space of wavelet coefficients. As an advantage over other clustering procedures, Combinato is more sensitive in the detection of clusters of small size (few action potentials). We visually inspected each identified cluster based on the shape and amplitude of the action potentials and the interspike interval (ISI) distributions. We removed clusters noisy waveforms, or nonuniform amplitude or shape of the action potentials in the recorded time interval. Moreover, to avoid overclustering, we merged highly similar clusters identified on the same microelectrode to obtain units. We considered only units with firing rate >1 Hz. Finally, we computed several metrics of spike sorting quality (Fig. 2b–d).”

## Data Records

The dataset was released in the G-Node/NIX format and can be downloaded at 10.12751/g-node.270z59^25,26^.The README describes the repository structure and the instructions for downloading the data.

Data from each participant was saved in a single hierarchical data format (.h5) file. Each file has the same format and includes general information, information on the task, participant and session, intracranial EEG data, spike times and waveforms, and information on depth electrodes (Table 2). We adhere to the standard NIX format. Whenever we introduce a custom name, we explain the name in NIX_File_Structure.pdf. The NIX_File_Structure.pdf describes the structure of that data that our script reads (Main_Load_NIX_Data.m). The script calls the NIX library and is well-commented.

**Table 2:** Data types in the NIX data.

General information	Institution conducting the experiment
	Recording location
	Related publications (name, doi)
	Recording setup (devices and settings)
Task	Name
	Description
	URL for downloading the task for Presentation^Ⓡ^
Participant	For each particpant: Age, gender, pathology, depth electrodes, electrodes in seizure onset zone (SOZ)
	Trials properties for each trial (trial number, condition, condition name)
Session	Number of trials
	Trial duration
Intracranial EEG data	For each trial: Bipolar montage of signals recorded with a sampling frequency of 2 kHz
	Labels and time axis
Spike waveforms	For each unit: Mean and standard deviation of spike waveform in a 2-ms window, sampled at 32 kHz
Spike times	For each unit: Spike time with respect to t = 0 in the trial
Depth electrodes	MNI coordinates in millimeters
	Electrode label
	Anatomical label updated after visual inspection of MRI
	Electrodes in the seizure onset zone (SOZ)

The dataset was also released in the iEEG-BIDS format on the OpenNeuro repository (10.18112/openneuro.ds003374.v1.1.1)^27–29^. This repository includes metadata and iEEG data, and also the extended dataset in the NIX format. iEEG data is provided as the BrainVision and European data format (EDF) files.

## Technical Validation

### Spike-sorting quality metrics

Spike sorting yielded single unit activity (SUA) and multiunit activity (MUA). We refer here to a putative unit by the term ‘neuron’. The example neuron in Fig. 2a increased its firing rate during the presentation of faces. For all neurons, the histogram of the percentage of inter-spike intervals (ISI) <3 ms is shown in Fig. 2b. The majority of neurons had less than 3% of short ISI. The percentage of ISI below 3 ms was 1.15 ± 0.9%. The histogram of average firing rate is given in Fig. 2c. The average firing rate of all neurons was 1.66 ± 2.65 Hz. For the mean waveform, the ratio of the peak amplitude to the standard deviation of the noise (waveform peak signal-to-noise ratio) was 4.62 ± 1.46 (Fig. 2d). These metrics are in the range of what is expected for the physiology of neuronal firing.

### Spectra of iEEG

**Fig. 3:**
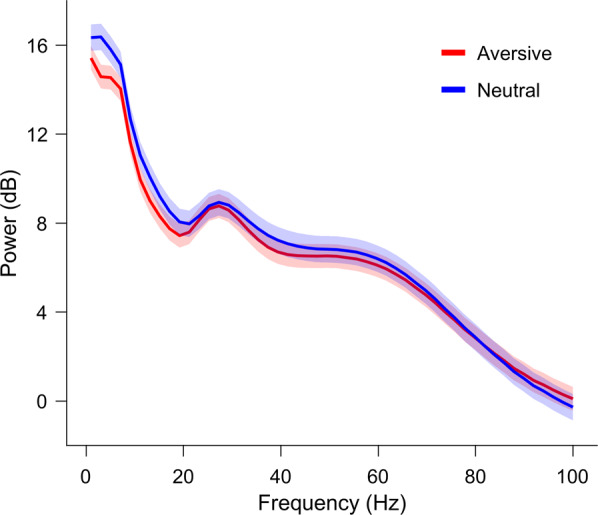
Power spectrum of the iEEG. The power spectrum for the aversive (red) and neutral (blue) conditions averaged over channels in the amygdalae outside of the SOZ (total 7 channels). Hann window 5 cycles of each frequency bin (temporal resolution: 100 ms; frequency resolution: 2 Hz).

iEEG power spectra of signals from healthy amygdalae (outside the seizure onset zone, Table 1) for the two conditions in Fig. 3.

## Data Availability

An example script is provided with the dataset^26,29^. It contains commented scripts for reading and plotting the data in NIX format^25^. We have also included scripts for the generation of Figs 2 and 3. All code is implemented in MATLAB (Mathworks Inc., version R2019a).
